# Trans Fatty Acids Induce Vascular Inflammation and Reduce Vascular Nitric Oxide Production in Endothelial Cells

**DOI:** 10.1371/journal.pone.0029600

**Published:** 2011-12-28

**Authors:** Naomi G. Iwata, Matilda Pham, Norma O. Rizzo, Andrew M. Cheng, Ezekiel Maloney, Francis Kim

**Affiliations:** Department of Medicine, Diabetes and Obesity Center of Excellence, University of Washington, Seattle, Washington, United States of America; University of Tor Vergata, Italy

## Abstract

Intake of trans fatty acids (TFA), which are consumed by eating foods made from partially hydrogenated vegetable oils, is associated with a higher risk of cardiovascular disease. This relation can be explained by many factors including TFA's negative effect on endothelial function and reduced nitric oxide (NO) bioavailability. In this study we investigated the effects of three different TFA (2 common isomers of C18 found in partially hydrogenated vegetable oil and a C18 isomer found from ruminant-derived—dairy products and meat) on endothelial NF-κB activation and nitric oxide (NO) production. Human endothelial cells were treated with increasing concentrations of Elaidic (*trans*-C18:1 (9 trans)), Linoelaidic (*trans*-C18:2 (9 trans, 12 trans)), and Transvaccenic (*trans*-C18:1 (11 trans)) for 3 h. Both Elaidic and Linoelaidic acids were associated with increasing NF-κB activation as measured by IL-6 levels and phosphorylation of IκBα, and impairment of endothelial insulin signaling and NO production, whereas Transvaccenic acid was not associated with these responses. We also measured superoxide production, which has been hypothesized to be necessary in fatty acid-dependent activation of NF-κB. Both Elaidic acid and Linoelaidic acid are associated with increased superoxide production, whereas Transvaccenic acid (which did not induce inflammatory responses) did not increase superoxide production. We observed differential activation of endothelial superoxide production, NF-κB activation, and reduction in NO production by different C18 isomers suggesting that the location and number of trans double bonds effect endothelial NF-κB activation.

## Introduction

Trans fatty acids (TFA) include monounsaturated fatty acids or polyunsaturated fatty acids that contain at least one carbon-carbon trans double bond. Most of the dietary TFA are derived from partial hydrogenation of vegetables oil or from ruminant-derived foods (dairy products and meat). TFA are widely used by the food industry in the generation of baked goods, deep-fried foods, and packaged snacks. It is estimated that TFA contribute up to 4–12% of total dietary fat intake in the US population [1]. During the past 20 years, epidemiologic studies have identified consumption of TFA as an important modifiable risk factor in the development of cardiovascular disease [2], [3]. Recently, the use and presence of TFA in the diet has been the object of much public health discussions.

Consumption of TFA raises levels of LDL cholesterol and reduces HDL cholesterol levels to a greater extent when compared with the consumption of equal amounts of energy from saturated or mono-or polyunsaturated fat. The relationship between intake of TFA and incidence of cardiovascular disease however, has been greater than predicted by changes in serum lipids alone, suggesting that TFA influence other risk factors for cardiovascular disease.

TFA consumption is known to influence multiple risk factors besides changes in lipid/lipoproteins, including increased systemic inflammation [4], increased thrombogenesis and reduced endothelial function [5], all of which in combination or individually contribute to increased cardiovascular risk. Experimental studies suggest that TFA exert their multiple effects by influencing metabolic and signaling pathways in hepatocytes, monocytes, adipocytes and in endothelial cells. The precise molecular pathways through which TFA influence these cell types are unknown.

Endothelial dysfunction can be described as impairment in the generation and function of nitric oxide (NO) as a vasodilator and vascular homeostatic agent. A reduction in NO bioavailability greatly increases the risk of developing atherosclerosis and hypertension. Endothelial nitric oxide synthase (eNOS) synthesizes NO in response to many agonists including fluid shear stress, bradykinin, and insulin, which increases NO production in endothelial cells through an Insulin Receptor Substrate-1 (IRS-1) and phosphatidylinositol 3-kinase (PI3-kinase) dependent pathway that results in phosphorylation of endothelial nitric oxide synthase (eNOS) by Akt in a calcium-independent manner [6], [7]. We have previously shown that the dietary saturated fat, palmitate, attenuates endothelial insulin signaling and NO production by first activating NF-κB signaling which results in a reduction in IRS-1/pAkt/peNOS signaling. Based on these results we asked whether the TFA would reduce endothelial NO levels by first activating NF-κB.

In endothelial cells, TFA have been shown to increase markers of endothelial dysfunction including E-selectin, ICAM, and impair flow-mediated vasodilation (measure of vascular NO production) in humans [5], [8]. In this study we chose three different TFA, all isomers of C18 – *trans*-C18:1 (9 trans), *trans*-C18:2 (9 trans, 12 trans), *trans*-C18:1 (11 trans). *Trans*-C18:1 (trans 9), also known as Elaidic acid, is a common TFA formed by partial hydrogenation of vegetable oil, *trans*-C18:2, also known as Linoelaidic acid, is commonly found during the process of heating vegetable oils (frying or baking food in vegetable oil) resulting in generation of two trans double bonds, *trans*-C18:1 (trans11), also known as Transvaccenic acid, is a model of ruminant-derived TFA, which are produced by bacteria in the ruminant stomach. Ruminant derived TFA are found in dairy products or in meat tissue, however human consumption of ruminant-derived TFA is generally low due to the low concentration (1%). All these TFA have 18 carbons, however differ in the location of the trans double bonds. We asked whether these different TFA are associated with differential effect on endothelial NF-κB and NO signaling.

## Methods

### Materials

Anti-phospho-IκBα antibodies were obtained from Cell Signaling (Beverly, MA) Human IL-6 ELISA kits from R and D Systems (Minneapolis, MN), and phospho-IκBα Case™ Cellular Activation of Signaling ELISA SuperArray Bioscience Corporation (Frederick, MD). Total Akt and pAkt(serine 473) ELISA kits were obtained from Biosource (Camarillo, CA). The spin trap 1-hydroxy-3-methoxycarbonyl-2,2,5,5-tetramethylpyrrolidine (CMH) was purchased from Alexis Biochemical (Lausen, Switzerland). Sodium DETC was obtained from Alexis Biochemical (Lausen, Switzerland). FeSO_4_ 7H2O was purchased from Sigma (St Louis, MO). Superoxide dismutase (PEG-SOD), Diphenylene idonium (DPI), and LPS were obtained from Sigma-Aldrich. Dihydroethidium (DHE) was purchased from (Molecular Probes/Invitrogen, Eugene Oregon).

Palmitic (C 16:0), C18:1T or 11-trans-octadecenoic (Transvaccenic), C 18:1T or 9-trans-octadecenoic (Elaidic), C18:2 (Linoleic), C18:2 Linoelaidic (9-trans 12 trans octadecadienoic) fatty acids were obtained from Nu-Chek Prep, Inc. (Elysian, MN) and BSA (FFA-free) was purchased from Roche (Indianapolis, In). FFA were dissolved in 0.1 M NaOH at 70°C and then complexed with 10% BSA at 55°C for 10 min to achieve a final fatty acid concentration of 100 µM as described previously [9]. Stock solutions of 5 mM FFA with 10% BSA and 10%BSA control solutions were prepared 1 d prior to experiments. Fatty acid preparations were assessed for LPS contamination using Amebocyte Lysate Test (Biowhittaker).

### Cell Culture

Human microvascular endothelial cells (HMEC) were purchased from (Invitrogen-Cascade Biological) and were cultured in RPMI 1640 supplemented with 10% fetal bovine serum (Hyclone Laboratories, Logan, UT) and 12 µg/ml of bovine brain extract (Clonetics, Walkersville, MD), L-glutamine (2 mM), sodium pyruvate (1 mM) and nonessential amino acids in the presence of penicillin (100 units/ml) and maintained at 37°C in 5% CO_2_. All Western bots were performed as described [10], using equal amounts of total protein for each condition and experiment. SDS gel electrophoresis was performed using a 4% by 20% gradient gel. Total RNA was extracted using RNeasy Mini Kit (Qiagen) and Human TNFα, ICAM, and iNOS primer pairs were purchased from Applied Biosystems.

### Superoxide measurement

Endothelial superoxide radical was measured by electron spin resonance spectroscopy (ESR) using the spin trap (CMH)[11], [12], [13]. HMEC were processed by washing once with ice cold PBS and removed by scrapping. After centrifugation the cells were resuspended in Krebs-HEPES buffer and 0.1 mM diethylenetriamine-penta-acetic acid (DTPA) was used to inhibit iron-catalyzed oxidation of the CMH trap. Electron spin resonance spectroscopy (ESR) studies were performed on a table-top x-band spectrometer Miniscope (Magnettech, Germany). Recordings were made at room temperature using a small capillary tube. Instrument settings were biofield 3350, Sweep 60 G, Microwave frequency 9.78 Ghz, microwave power 20 mW, and kinetic time of 10 min.

### Nitric Oxide Measurements

Nitric oxide was measured using the spin trap Fe(DETC)_2_ which was first reported and validated by Kleschyov et al [14]. Preparation of colloid Fe(DETC)_2_: Sodium DETC (3.6 mg) and FeSO_4_ 7H20 (2.25 mg) were dissolved under argon gas in 10 ml of ice cold Krebs-Hepes buffer [15] (consisting of, in mM: NaCl 99, KCl 4.7, MgSO_4_ 1.2, KH_2_PO_4_ 1.0, CaCl_2_ 1.9, NaHCO_3_ 25, glucose 11.1, and Na-Hepes 20, pH7.4) These were rapidly mixed to obtain a pale yellow-brown colored Fe(DETC)_2_ solution which was used immediately. After removing cell culture media, endothelial cells were washed once with PBS and 100 µl of Krebs-hepes buffer was added to the cell culture plates. Colloid Fe(DETC)_2_ was then added to final concentration of 286 µM and incubated at 37°C for 90 minutes. Electron spin resonance spectroscopy (ESR) studies were performed on a table-top x-band spectrometer Miniscope (Magnettech, Germany). Recordings were made at 77 K using a Dewar flask. Instrument settings were 10 mW of microwave power, 1 mT of amplitude modulation, 100 kHz of modulation frequency, 20 s of sweep time and 10 number of scans.

### Statistics

In all experiments, densitometry/ELISA measurements were normalized to controls incubated with vehicle and percent change relative to the control condition was calculated. Analysis of the results was performed using the STATA8 statistical package. Data are expressed as mean ± SEM, and values of p<0.05 were considered statistically significant. A two-tailed *t*-test was used to compare mean values from studies involving two experimental groups. Data were analyzed by two-way analysis of variance using the Bonferoni-post-hoc comparison test when appropriate.

## Results

### Trans-fatty acids induce NF-κB responses in human endothelial cells

**Figure 1:**
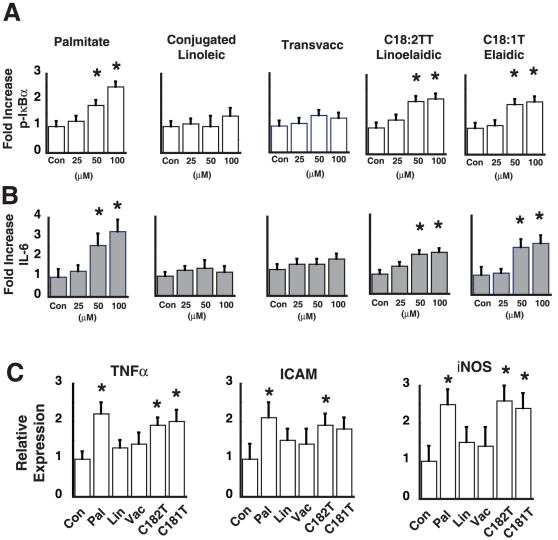
Effect of TFA on endothelial NF-κB signaling. Human endothelial cells were treated with increasing concentrations of palmitate, linoleic acid, transvaccenic (*trans*-C18:1 (11 trans)), linoelaidic (*trans*-C18:2 (9 trans, 12 trans)), and elaidic (*trans*-C18:1 (9 trans)) for 3 h. All fatty acids were initially complexed with BSA and in the control condition cells were treated with BSA alone for 3 h. **A**. Phospho-IκBα levels were measured using an ELISA assay. Fold-increase over the control (BSA alone) condition was calculated. (n = 3, *p<0.05). **B**. IL-6 levels as measured by ELISA. (n = 3, * p<0.05). **C**. Expression levels of TNFα, ICAM, and iNOS in response to 100 µM of fat. (n = 3, * p<0.05).

We first asked whether TFA, found in a Western diet, are associated with increased endothelial NF-κB activation. We tested three different TFA: *trans*-C18:1 (11 trans), *trans*-C18:2 (9 trans, 12 trans) and *trans*-C18:1T (9 trans) all different isomers of C18. We used palmitate as a control since we have previously shown that palmitic acid increases endothelial NF-κB activation in a dose dependent manner [16]. C18:2 in the cis configuration (linoleic) was also used as a negative control since this polyunsaturated fat does not increase endothelial NF-κB signaling. We chose an incubation period of 3 h with BSA-complexed fats, since previous studies demonstrated an apoptotic effect in endothelial cells for time periods greater than 6–8 h. To assess NF-κB activation in endothelial cells we measured the phosphorylation of IκBα and production of IL-6 (NF-κB dependent cytokine). Both *trans*-C18:2 (9 trans, 12 trans) and *trans*-C18:1 (9 trans) increased phospho-IκBα and IL-6 levels in a dose dependent manner, whereas C18:2 (Linoleic) and *trans*-C18:1 (11 trans) (Transvaccenic acid) did not increase phospho-IκBα or IL-6 levels (Figure 1A, B). As expected, palmitate increased NF-κB signaling in a dose-dependent manner (Figure 1). Both Elaidic (*trans*-C18:1 (9 trans)) and Linoelaidic (trans-C18:2 (9 trans, 12 trans)) at concentration of 100 µM, increased NF-κB-dependent gene expression (TNFα, ICAM, and iNOS), a response not seen with Linoleic or Transvaccenic acid (Figure 1C). Thus, common “industrial TFA” are associated with increased endothelial inflammation, whereas ruminant derived TFA (transvaccenic acid) does not. Furthermore, the location of the trans-double bond had a differential inflammatory effect on endothelial cells.

### Effect of trans fats on endothelial insulin signaling

**Figure 2:**
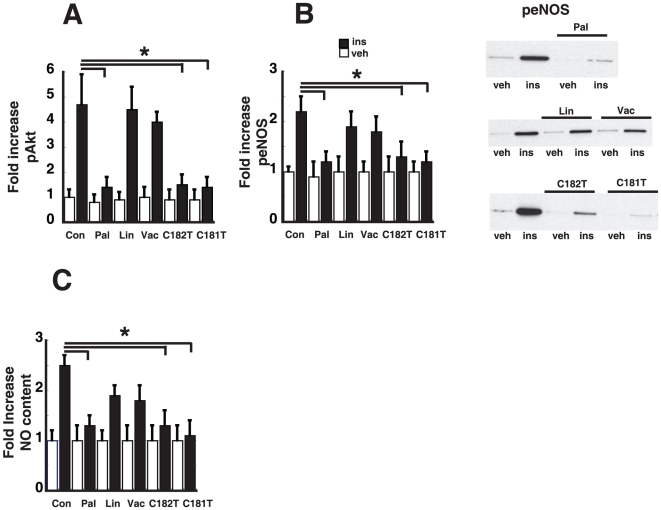
Effect of TFA on endothelial insulin signaling. Human endothelial cells were treated with either 100 µM of palmitate, linoleic acid, transvaccenic (*trans*-C18:1 (11 trans)), linoelaidic (*trans*-C18:2 (9 trans, 12 trans)), and elaidic (*trans*-C18:1 (9 trans)) for 3 h and then stimulated with insulin 100 nM or vehicle for 15 min. **A**. Cell lysates were made and were analyzed for insulin-mediated phosphorylation of serine 473 Akt and total Akt by ELISA. Fold increase over vehicle condition was calculated. (n = 3, *p<0.05) **B**. Cell lysates were analyzed for insulin-mediated phosphorylation of serine 1177 eNOS and total eNOS by Western blot. Representative phospho-eNOS Western blots are shown. Fold increase over vehicle condition was calculated (n = 3, *p<0.05). **C**. Insulin-mediated nitric oxide production was measured by ESR using the spin trap Fe(DETC)_2_ (n = 3, *p<0.05).

We have previously shown that in endothelial cells, palmitate-mediated activation of NF-κB signaling is associated with reduced insulin-mediated Akt and eNOS signaling in endothelial cells [16]. We next investigated whether TFA would also reduce endothelial insulin signaling. Endothelial cells were treated with 100 µM of TFA for 3 h and insulin-mediated signaling was assessed following treatment with vehicle or 100 nM insulin for 15 minutes. As expected and consistent with published results, pre-treatment with 100 µM of palmitate resulted in reduced insulin-mediated Akt and eNOS phosphorylation in the absence of any change in total eNOS or Akt levels. Although insulin stimulation increases phosphorylation of Akt serine 473 and eNOS serine 1177, pretreatment with *trans*-C18:2 (9 trans 12 trans) and *trans*-C18:1 (trans 9) are associated with impairment of endothelial insulin signaling, however, we did not see an inhibitory effect with *trans*-C18:1(11 trans) (Transvaccenic acid) or with Linoleic acid (Figure 2A and 2B). TFA, which are associated with increased NF-κB activation are also associated with reduced endothelial insulin signaling.

### Effect of TFA on NO production

We next determined the effect of TFA on endothelial NO production as measured by ESR using the spin trap Fe(DETC)_2_, an established and sensitive method for NO detection [17], [18]. As expected palmitate pretreatment is associated with reduced insulin-mediated NO production (Figure 2C) whereas pretreatment with Linoleic acid (C18:2) or transvaccenic acid did not reduce insulin mediated NO production. TFA associated with increased endothelial NF-κB activation (*trans*-C18:2 and *trans*-C18:1) are also associated with reduced insulin-mediated NO production, an expected result since we have previously linked NF-κB activation with a reduction in NO production.

### Effect of TFA on superoxide production

**Figure 3:**
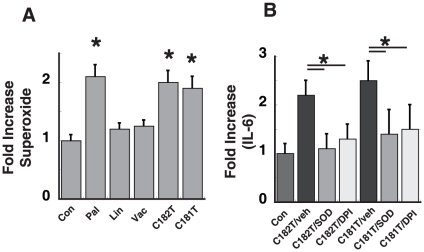
Effect of TFA on endothelial superoxide production. Human endothelial cells were treated with either 100 µM of palmitate, linoleic acid, transvaccenic (*trans*-C18:1 (11 trans)), linoelaidic (*trans*-C18:2 (9 trans, 12 trans)), and elaidic (*trans*-C18:1 (9 trans)) for 3 h. **A**. Superoxide levels were measured by ESR and the spin trap CMH. Fold increase over the control condition (BSA alone) was calculated. (n = 3, *p<0.05, compared to BSA control). **B**. Cells were pretreated with either of two inhibitors of superoxide (SOD, 100 Units/ml or DPI 25 µM) prior to stimulation with Elaidic or Linoelaidic acid. IL-6 levels as measured by ELISA.

In previous studies, palmitate increases NF-κB activation via a mechanism dependent on NADPH oxidase-derived superoxide production [19]. We next asked whether TFA would also increase reactive oxygen species (ROS) production. Endothelial cells were treated with 100 µM of TFA and ROS production was measured by ESR and the spin trap CMH. As expected 100 µM of palmitate increased ROS production 2-fold, whereas Linoleic acid and Transvaccenic acid did not increase ROS production. *Trans*-C18:1 (9 trans) and *trans*-C18:2 (9 trans, 12 trans) were associated with a 2-fold increase in ROS production as measured by ESR. TFA, which are associated with increased endothelial NF-κB are also associated with increased ROS production. Pretreatment with superoxide dismutase (SOD) or incubation with diphenylene iodnium (DPI) attenuates palmitate-dependent activation of endothelial inflammation [19] and as expected, similar pretreatment attenuates activation of endothelial NF-κB (at the level of IL-6 production) in the presence of *trans*-C18:1 (9 trans) and *trans*-C18:2 (9 trans 12 trans), suggesting that these TFA also activate NF-κB via a mechanism dependent on ROS production (Figure 3B).

## Discussion

Numerous epidemiological studies have correlated elevated dietary intake of TFA with increased mortality and morbidity from cardiovascular disease. In this study we examined three TFA for their ability to activate endothelial NF-κB and subsequently reduce NO production. We found that two of the TFA, *trans*-C18:2 (9 trans 12 trans)(Linoelaidic) and *trans*-C18:1 (9 trans) (Elaidic) are associated with increased NF-κB activation, and reduced endothelial insulin signaling and NO production, whereas, *trans*-C18:1 (11 trans)(Transvaccenic) is not associated with either of these responses. Furthermore, both Linoelaidic and Elaidic acids increased endothelial ROS production, a response, hypothesized to be necessary in activating endothelial NF-κB. These results suggest that different isomers of C18 are capable of eliciting differential effect on endothelial function.

Exposure of endothelial cells to palmitate, glucose, TNF-α all mediators of insulin resistance, results in activation of NF-κB and increased expression of adhesion molecules [20], [21], an early marker for atherosclerosis. In addition, we have shown that these mediators of insulin resistance are also associated with reduced endothelial NO production [6], [22]. Raising systemic inflammation by injection of S. typhi vaccine has been shown to impair endothelial function in human volunteers [23], suggesting that systemic inflammation effects nitric oxide bioavailability. The mechanism underlying endothelial dysfunction may therefore involve activation of NF-κB, which by inhibiting IRS-1/PI3-kinase signal transduction, attenuates NO production. Attenuation of endothelial NF-κB signaling (using a mutant of IκBα) in the presence of palmitate restores endothelial NO content [24], which further supports a mechanistic link between NF-κB and reduced NO levels. In the present study, TFA which are associated with increased NF-κB signaling are associated with reduced endothelial NO signaling. The relationship between *trans*-C18:1 (Elaidic)and *trans*-C18:2 (Linoelaidic) on endothelial NF-κB activation have been demonstrated before [8], and our studies are in support of the previous observation. However, we extend this finding by linking NF-κB activation with reduced endothelial insulin signaling and NO production.

The relationship between total industrial TFA (partially hydrogenated vegetable oil) consumption and cardiovascular disease has been well studied. Less is known, however, about the vascular effects of particular TFA isomers. During the partial hydrogenation of vegetable oil (industrial TFA) such as *trans*-18:1, the location of the double bond vary from carbon number 4 to 16, however, the double bonds are mainly centered on carbon 9 or 10. The distribution depends first on the starting vegetable oil and secondly, on the extent of hydrogenation. Whether a particular *trans*-C18:1 isomer has a greater cardiovascular risk remains an unanswered question, however, maybe difficult to answer using population based studies. Our current in vitro studies, however, support the hypothesis that the different *trans*-C18:1 isomers have a differential effect on endothelial NF-κB activation and on NO and superoxide production. Furthermore, these differential effects may serve as “mechanistic” explanations for differential clinical risk of the different TFA isomers and suggests that some C18:1 isomers (eg. Transvaccenic acid) may have limited effects on endothelial cells.

The effect of ruminant derived TFA (eg. Transvaccenic acid) on cardiovascular disease risk or endothelial dysfunction have not been clearly defined. Tardy et al. recently reviewed 5 studies, which looked for an association between consumption of ruminant derived TFA and cardiovascular risk [25]. In contrast to the industrial TFA, no conclusive evidence is currently available which links ruminant derived TFA with cardiovascular risk. Ruminant derived TFA may be less deleterious especially at lower level and consumption of dairy fats could be tolerated in respect to cardiovascular risk. Furthermore, no specific relationships between ruminant TFA and systemic inflammation or endothelial dysfunction have been defined in small observational studies. Our current in vitro studies with Transvaccenic acid (our model of ruminant derived TFA) support this observation and are consistent with the observed clinical studies.

The precise molecular pathways by which TFA increase NF-κB signaling remains unanswered, however a few possible mechanisms have been proposed. First, many dietary fats are known to incorporate into cell membrane and lipid rafts, which may alter cell receptor function. In vitro studies in human aortic endothelial cells demonstrated increased incorporation of *trans*-C18:2 into cellular membrane, which was associated with increased expression of adhesion molecules [8]. Second, TFA may alter fatty-acid metabolism by reducing fatty acid uptake and esterification. Third, our laboratory and others have shown a role for the innate immune receptor, toll like receptor 4 in mediating the effects of saturated fat in activation of NF-κB [24]. It is possible that TFA may directly activate TLR4 or indirectly by altering lipid raft function. Finally, our laboratory and others have demonstrated a potential role for NADPH oxidase-dependent ROS generation in mediating the inflammatory effects of dietary fats [19]. Reduction of NAPDHoxidase signaling or ROS generation attenuates the ability of palmitate to increase NF-κB signaling. In the current study we also observed a correlation with the generation of ROS and endothelial NF-κB activation.

Clinical studies have demonstrated a profound effect of FFA on NO production. In normal patient volunteers, the ingestion of a single high-fat meal transiently impairs endothelial function as measured by flow-mediated brachial artery vasodilation [26]. Infusion of high doses of intralipid plus heparin into normal volunteers raises circulating FFA concentrations from a starting concentration of 350 µM to a peak of 3800 µM [27]. Methacholine-induced vasodilation was reduced by as much as 20%, indicating that elevated FFA levels induce endothelial dysfunction [27]. In a separate study, raising FFA levels resulted in impairment of basal and insulin-mediated NO production [28]. These human studies suggest that the production of NO is impaired in the presence of high circulating levels of FFA.

We used doses of individual TFA between 25–100 µM in our in vitro studies, which are higher than found in humans. A diet rich in various *trans*-C18:1 has been shown to increase the serum levels of all TFA to between 40–120 mM [29], suggesting that the in vitro study TFA doses maybe 2–4 times higher than found in human serum. In vitro studies are limited in that higher concentrations are necessary in order to observe a biologic effect and need to be confirmed in dietary studies.

In conclusion, the present studies demonstrate that two common industrial TFA isomer of C18 increase NF-κB activation and impair insulin-mediated NO production in endothelial cells. These studies suggest a differential effect of C18 isomers and that the location and number of trans double bonds effect endothelial NF-κB activation. Furthermore, these studies provide mechanistic insight into the role of TFA in mediating endothelial dysfunction leading to vascular disorders.
